# The citizen perspective on challenges and rehabilitation needs among individuals treated for head and neck cancer: a qualitative study

**DOI:** 10.1007/s00520-025-09163-9

**Published:** 2025-01-28

**Authors:** Kathrin Fríðunn Astrup Brøgger Jacobsen, Lene Kronborg Mikkelsen, Lone Jørgensen

**Affiliations:** 1 Aalborg Health Center and Rehabilitation, Aalborg Municipality, Denmark; 2 Specialist Centre for Adult Dental Care, Aalborg Municipality, Denmark; 3 Clinical Nursing Research Unit, Aalborg University Hospital & Department of Clinical Medicine, Aalborg University, Aalborg, Denmark

**Keywords:** Head and Neck cancer, Rehabilitation needs, Citizen perspective, Qualitative research

## Abstract

**Purpose:**

In Denmark, the prevalence of head and neck cancer is approximately 17.000, and the incidence is increasing. The disease and treatment of this condition may lead to severe physical, psychological, and social consequences. However, the literature indicates a lack of rehabilitation services and insufficient professional resources in the municipal setting resulting in unmet rehabilitation needs. The aim of this study is to gain an understanding of the challenges and rehabilitation needs experienced by citizens treated for head and neck cancer.

**Methods:**

A qualitative study using semi-structured interviews was employed. Paul Ricoeur’s interpretation theory was used to analyze the data.

**Findings:**

Citizens treated for head and neck cancer experience the need for targeted assistance to manage the consequences following treatment for head and neck cancer and the need for adequate information and specialized professional competencies in municipal rehabilitation. The findings highlight a dual need: support from healthcare professionals and opportunities for patients to connect with others who have undergone treatment for head and neck cancer, as part of municipal rehabilitation.

**Conclusion:**

The study contributes to an understanding of the citizen perspective on rehabilitation needs and informs and enhances knowledge about municipal rehabilitation interventions for citizens treated for head and neck cancer. However, the findings also indicate the complexity of the referral process, highlighting the need for further research on barriers and facilitators to referral and access to municipal rehabilitation.

## Introduction

Head and neck cancer (HNC) constitutes a significant health burden, primarily caused by smoking, alcohol consumption, and Human Papilloma Virus (HPV) [1]. Alcohol and smoking are associated with up to 72% of HNC cases, while HPV-related HNC is increasing [2]. Furthermore, occupational exposure, previous radiation therapy, diet, genetics, poor oral hygiene, and periodontal disease are related to HNC [3–5]. Studies also indicate higher risks of developing HNC with decreasing socioeconomic status (SES) [6, 7]. In Denmark, HNC is on the rise with a prevalence of around 17,000 cases and many are diagnosed in stages 3 and 4, requiring more aggressive treatment and extensive rehabilitation [8]. Cancer in the pharynx and oral cavity constitutes most cases and the treatment depends on the type of cancer, its location, and the stage of the disease and may include surgery, radiation therapy, and in certain cases, chemotherapy [8, 9]. The consequences vary in severity and may include eating and swallowing difficulties (dysphagia) and dry mouth (xerostomia), impacting nutrition and dental health [10], which increase the risk of weight loss, associated with increased mortality [11]. Other consequences such as mucositis, osteoradionecrosis, and physical dysfunctions may also arise and negatively affect quality of life [12]. Furthermore, the treatment can lead to psychosocial challenges such as anxiety, depression, and a diminished self-image [13]. Rehabilitation efforts are crucial in addressing these challenges encompassing the physical, psychological, and social aspects through multidisciplinary collaboration [9, 14]. However, it is problematic that only 20% of Danish municipalities offer specialized rehabilitation to citizens treated for HNC, along with a general lack of the necessary expertise [15, 16], a tendency that is also indicated in international research [17]. This is particularly problematic as citizens treated for HNC with low SES are less likely to be referred to or participate in rehabilitation programs [18].

In Denmark, there is a growing recognition of the need for enhanced rehabilitation for citizens treated for HNC, and studies indicate that rehabilitation should integrate the citizen’s perspective to meet individual rehabilitation needs to develop rehabilitation programs tailored to citizens treated for HNC [19–21]. Therefore, this study aims to explore the perceived needs for rehabilitation among citizens treated for HNC in Denmark.

## Methods

### Design

This study has a qualitative design using a phenomenological-hermeneutic approach with semi-structured interviews to explore the perceived need for rehabilitation among citizens treated for HNC. The Consolidated criteria for Reporting Qualitative research (COREQ) checklist was used as a guideline for reporting the study [22].

### Sampling

Convenience sampling was used to recruit participants with different characteristics such as variation in age and gender [23]. Recruitment was conducted over 14 days in April 2023. Healthcare professionals (HCP) in a rehabilitation unit and health center in a Danish municipality as well as HCP’s in an oncology department at a Danish university hospital assisted with the recruitment of participants. The inclusion criteria were participants with HNC, including cancer in the oral cavity, tonsils, lips, and throat, as well as the larynx, thyroid gland, sinuses, middle ear, and nasal cavity. In Denmark, municipal rehabilitation is a free service provided to citizens who have been treated for HNC. Participants were included regardless of whether they had been referred to or had participated in municipal rehabilitation interventions. Exclusion criteria were citizens with cognitive impairment or unable to give informed consent, or unable to speak or understand Danish.

### Setting

The interviews were conducted by two female authors, (KJ, LM) studying a master’s degree in public health and with no prior knowledge of or relation to the participants. The interviews took place either in the participants’ home or by telephone depending on their preference. In total, 10 participants were interviewed. At the informant’s request, six were interviewed in their home by one author, but with both authors being present due to sparse experience with interviewing. To manage inexperience, the telephone interviews were conducted after the in-person interviews. One of the authors acted as the primary interviewer while the other author was responsible for asking follow-up questions. Four participants chose to be interviewed by phone and the interviews were conducted by one author, with the interviews evenly distributed between the two authors (KJ, LM). The duration of each interview was approximately 45–60 min.

### Data collection

A semi-structured interview guide (Table 1) was developed based on the aim of the study and reviewed thoroughly (KJ, LM, LJ) before being validated in a pilot interview with a HCP with HNC rehabilitation experience. Data were collected over the course of a week in April 2023, and the interviews were audio recorded and verbatim transcribed (KJ, LM) using a transcription guide.

**Table 1:** Examples of questions from the interview guide

Questions	Exploratory questions
Can you tell me about the impact the disease has had on your everyday life?	Can you give some examples of how the disease affects your daily activities?
How would you describe the needs you have to return to your everyday life?	What do you feel it takes for you to return to your everyday life? Can you tell me more about that?

### Data analysis

Data were analyzed according to Ricoeur’s interpretation theory inspired by Pedersen and Dreyer [24]. The analysis contained a naive reading, structural analysis (what is said), structural analysis (what the text speaks about) and then critical interpretation and discussion. In the naive reading, the entire data material was read openly, and impressions were written down. The structural analysis acted as the link between surface interpretation (meaning units) and depth interpretation (condensed meaning units) and themes were derived from the analysis of the condensed meaning units. Critical interpretation, a movement toward discussion and in-depth understanding occurred using research literature and theory [24]. This created a horizon merging between the text and the understanding of the text [24] and contributed to the understanding and explanation of the perceived need for rehabilitation among citizens treated for HNC.

### Ethical considerations

The study was based on ethical guidelines formulated in the Helsinki Declaration to ensure the voluntariness of participation, as well as to safeguard the privacy and confidentiality of data [25]. Pseudonyms were used to ensure anonymity. No approval has been applied for from the Scientific Ethics Committee, as it is not a requirement in Denmark. Informed consent was obtained orally or in writing. Files and data were stored on an encrypted hard disk and deleted after completion of the study in accordance with current regulations [26].

### Findings

A total of 10 participants participated in the qualitative interviews (Table 2).

**Table 2:** Participant characteristics (*n* = 10)

Participant	Gender	Age	Diagnosis	Time between diagnosis and interview	Recruited from	Participated in municipal rehabilitation
1	Woman	60s	Oral cancer	8 months	Municipal	Yes
2	Man	60s	Tonsil cancer	6 months	Municipal	Yes
3	Woman	50s	Tonsil cancer	4 months	Municipal	Yes
4	Man	60s	Cancer of the lower pharynx and esophagus	4 months	Municipal	Yes
5	Woman	50s	Cancer of the root of the tongue	4 months	Municipal	Yes
6	Woman	60s	Esophageal and thyroid cancer	6 months	Municipal	Yes
7	Woman	30s	Cancer of the nose	13 months	Region	No
8	Man	30s	Tonsil cancer	5 months	Region	Yes
9	Man	80s	Cancer of the lower pharynx and larynx	5 years and 2 months	Region	No
10	Man	50s	Cancer of the root of the tongue	5 years	Region	Yes

### The naive reading

The participants experienced significant challenges in the post-treatment period, impacting their physical and mental well-being, daily activities, and social lives. Issues such as pronounced fatigue, difficulty in eating and swallowing, and pain during consumption of food and drinks were highlighted, posing a significant challenge that also restricted social interactions, as it felt intrusive to eat with others. While municipal rehabilitation in managing these challenges was deemed sufficient, the participants felt a pressure from the HCP’s to avoid weight loss, with a predominant focus on weight and nutrition rather than a holistic understanding of the difficulty in eating and gaining weight. Despite being informed about treatment side effects, some felt adequately prepared, while others were overwhelmed. The participants also expressed a need for support from others who had undergone similar treatment, emphasizing the value of sharing experiences with those who could empathize and understand their situation.

### Structural analysis

In the structural analysis selected quotes are expressed as meaning units. Based on what is being talked about in the meaning units, the condensed meaning units were formed. In total, 108 meaning units and 24 condensed meaning units were identified. In the analysis, three themes were identified: (1) “The complexity of rehabilitation needs and the challenge of accessing necessary rehabilitation,” (2) “The complex diversity of varying information needs and the balance of targeted information,” and (3) “The psychological consequences and the need for support and understanding” (Table 3).

**Table 3:** An excerpt from the structural analysis

Meaning units	Condensed meaning units	Themes
“Well, just eating a small egg, or I don’t know. It wasn’t an egg. It was some scrambled something. Well, it took half an hour to sit and struggle with it. I mean, that made you upset.” (I1)	Eating and swallowing difficulties constitutes a significant limitation for ingestion of food and drink and limits the social life	The complexity of rehabilitation needs and the challenge of accessing necessary rehabilitation
“The thing is, just the act of eating and being together with other citizens and enjoying oneself. You can’t do that in the same way. It’s embarrassing..” (I1)		
“I mean because they push so hard, right, and they have no understanding of what you’re going through. I know it’s important to maintain your weight. But it doesn’t help forcing citizens so much that they throw up as soon as they’ve had the tube feed.” (I5)	Lack of understanding and demands from HCP regarding nutritional challenges create frustration	
“I knew it was a significant treatment, but I was not aware it was this substantial and would change so much for my life going forward.” (I1)	Being unprepared for the duration and severity of side effects and uncertainty about how side effects are managed	The complex diversity of varying information needs and the balance of targeted information
“I found it a bit overwhelming, and I know that they, of course, need to inform about all possible or potential side effects, but I thought it was an overwhelming experience to have so many things outlined that could potentially happen.” (I7)	Information about side effects is overwhelming.	
“So mentally, I live with the anxiety every day. I do. I mean, even if there’s the slightest thing, I think: ‘No, now something is wrong.’ I do. And I’m probably more sensitive there because I’m so afraid that something will happen again. Really anxious.” (I1)	The fear of the disease returning has psychological consequences	The psychological consequences and the need for support and understanding
“Well, I think there is a huge value in that because it’s on a level where it makes sense, where you are with peers. So, you’re not alone in the world. There are actually citizens right next to us who are thinking some of the same thoughts.” (I10)	Contact with others treated for head and neck cancer holds value in terms of support and understanding	
“So, I have chosen to seek outside, bypassing the municipality, and independently pursue psychological counseling. (...) because I could feel that it was a state of shock that (F) probably needed to process together with someone who knew more than I did. So, I think that’s the only thing where I’ve thought that, yes, it’s a service that I have missed and sought.” (I7)	The psychological consequences of the disease create a need for professional help and support	

## Critical interpretation and discussion

### Theme 1. The complexity of rehabilitation needs and the challenge of accessing necessary rehabilitation

The findings highlighted the persistent consequences of treatment for HNC, where eating and swallowing difficulties constituted significant challenges in consumption of food and drink. The findings supported previous research showing that sequelae may persist for a long period of time [27–29]. These challenges underscore the importance of rehabilitation efforts addressing eating and swallowing difficulties to maintain adequate nutrition and prevent weight loss. Weight loss does not exclusively have a negative impact on quality of life [10] but can also be linked to an increased risk of mortality [11], which shows the importance of addressing the nutritional challenges. Our findings support existing evidence of how difficult people living with HNC find maintaining adequate nutrition and weight during treatment and the subsequent burden of HCPs lack of understanding of this:

“But she (HCP) was really difficult to deal with, and it’s like they don’t quite get it. I know the thing about weight. I’m not stupid, right. But the fact that you’re constantly being told that you must get it (the weight) up. Well, I know, what the hell am I going to do? I can’t stop it, can I? Well, when I vomit, I vomit.” (I5)

This highlights the tension some citizens experience when struggling to maintain nutrition and weight during treatment. While HCPs emphasize the importance of weight maintenance, often due to clinical considerations such as mask fit and treatment precision [30], this can sometimes be perceived as overlooking the emotional and physical challenges patients face. The findings also demonstrated that eating and swallowing difficulties limit social life, as meals in social contexts were often perceived as embarrassing, leading to isolation. This confirms other research findings where these difficulties were hidden from others as they evoked the fear of feeling weird, restricting socialization [27, 31]. Other literature indicates that optimal oral hygiene after treatment for HNC can help improve eating and swallowing difficulties [32, 33]. In the rehabilitation process, optimal oral hygiene is essential, not only for the extent of eating and swallowing difficulties but also for participation in social life. Targeted information, instruction, and ongoing follow-up about optimal oral hygiene by trained HCPs support oral health in individuals [34], indicating that continuous follow-up about oral hygiene should be an integral part of rehabilitation efforts.

Our study showed that the time after treatment was characterized by pain, limiting the intake of food and drink. This is supported by previous literature where pain also challenged everyday life [28]. According to the Danish “Head and Neck Cancer Follow-up Program,” medical treatment is necessary in rehabilitation efforts to reduce pain [9]. However, the literature indicates that in addition to pain treatment, physiotherapy, training, swallowing exercises, and psychosocial intervention after treatment must be offered to reduce pain [34, 35]. A combination of medical treatment and other forms of treatment should therefore be an integral part of rehabilitation interventions. In our study, sleep disturbances and pronounced fatigue increased the need for sleep, limiting participation in meaningful everyday activities:

“Well, it has meant a lot because I can’t do the same things as I used to (...) I’m very tired, especially on those days when I vacuum or do things like that, I get tired. Then I can easily sleep like 3–4 times during the day.” (I3)

Previous research has shown that physical exercise can reduce fatigue among citizens treated for HNC [36]. Rehabilitation efforts should therefore address individual causes of sleep disorders and include physical training in an individualized approach to managing this problem.

Findings from our study showed examples of no referral to rehabilitative efforts despite physical, mental, and social needs due to lack of clarity about financial matters in the municipality, and uncertainty about the responsibility for referrals. The consequences are delay in rehabilitation and lack of help in dealing with consequences such as pain, excessive fatigue, poor nutritional status, weight loss, reduced quality of life, and social isolation [10, 37]. Furthermore, lack of rehabilitation causes unnecessary negative unintended consequences at community level such as increased sickness absence and equivalent productivity loss and increased disease burden and mortality [38, 39]. Limited access to rehabilitation increases the risk of social inequalities in health, as citizens with low SES face greater challenges in accessing and participating in rehabilitation interventions [18]. This indicates that communication and clarity about responsibilities and the financial framework for referral to municipal rehabilitation services should be improved to ensure a well-coordinated, coherent, and timely effort in municipal rehabilitation.

### Theme 2. The complex diversity of varying information needs and the balance of targeted information

Information about side effects or sequelae to the treatment of HNC was not sufficient to prepare the participants for the duration, severity, and significant changes in everyday life after treatment:

“They (the consequences) have been terrible. It’s really been, I hadn’t imagined at all. I’m a pretty tough type, but I must say, I’ve become a bit of a wimp after this.” (I4)

Previous research confirms that insufficient information and lack of preparation are critical [40], as a significant portion of participants consider information about consequences and prevention important to manage side effects [27–29]. Respectful and clear communication from HCP is valued as crucial to meet the need for information and the opportunity to ask questions [39]. However, excessive information can be overwhelming:

“I think they were very comprehensive and told me about the worst-case scenario. And that’s fair enough too. But I wasn’t (...) able to, like (...) take it in, you know. I couldn’t relate to it.” (I10)

The consequence of information overload can be challenges in handling, understanding, and translating information [19], which indicate differences in the need for information. Varying information needs are known [41] and may also be related to health literacy and quality of life [40]. Furthermore, varying preferences for how and when information is presented are seen among citizens treated for HNC, and up to 25% wish to receive information more than once [42]. This underscores the importance of HCP striving to identify individual information needs and target the amount of information accordingly as emphasized in international guidelines for management of HNC [43].

Our findings also demonstrated the need to discuss disease-related knowledge with HCP with specialized knowledge in HNC, which is supported in other research findings [28, 29, 41, 42]. However, there is a tendency toward a lack of specialized expertise among HCP in municipal rehabilitation [16], which can affect the quality of the information and rehabilitation provided. Therefore, the availability of specialized HCP’s needs to be considered in the rehabilitation process to meet individual information needs.

### Theme 3. The psychological consequences and the need for support and understanding

Findings from our interview study indicated a feeling of being changed after treatment for HNC as well as an experience of not being understood by family and friends.

“I also sometimes feel that some people don’t quite understand it. Because it’s hard to see. It’s hard to feel. You also can’t see how I feel inside. (NV) (...) There is an understanding of it, but still, I don’t think people really get it. (...) I mean, we can always say: ‘Well, I understand you,’ but you don’t, not until you’ve... You can only understand it by experiencing it yourself.” (I1)

This quote illustrates that relating to the challenges faced by survivors of head and neck cancer treatment is difficult without personal experience. However, our findings show that personal contact with others treated for HNC can provide access to the support and understanding that citizens do not always experience in their closest relationships:

“Well, there is, I think, a huge value, because it’s on a level where it makes sense, where you’re together with peers. So, you’re not alone in the world. There are actually some citizens right next to us who are thinking some of the same thoughts.” (I10)

Contact with other peers are found to be significant, as sharing experiences among patients treated for HNC can provide valuable understanding [27, 29] and reduce the feeling of uncertainty, loneliness, and social isolation [44]. Our findings therefore indicate that conditions for sharing experiences should be supported in municipal rehabilitation. In this context, other research highlights that peer support should be organized focusing on citizens’ individual preferences as well as gender and age [44]. Furthermore, our findings show that the feeling of understanding can also be gained through written accounts without personal contact. It is therefore important to facilitate access to information about others’ experiences in the municipal rehabilitation, however, without replacing professional counseling [44]. This is significant because our findings demonstrated that care and empathy in contact with HCP contribute positively to the experience of emotional support:

“The fact that I could talk to someone, someone who took care of me. So, the care you suddenly experience... I know it’s professional, but still, I feel empathy” (I1)

The importance of professional empathism is also confirmed in other studies [28]. Findings in our study also suggest a need for psychological support tailored to individual needs:

“So, I chose to seek outside the municipality, and seek psychological counseling on my own. (...) because I could feel that it was a state of shock that needed to be processed with someone who knew more than I did.” (I7)

This quote highlights a lack of adequate and individualized psychological support in the municipality’s rehabilitation services, leading to seeking help outside the municipality. Prioritizing psychological support in municipal rehabilitation can not only address the psychological consequences but also aid in quicker return to work, as psychological issues are a leading cause of sick leave post-HNC treatment [38], leading to increased productivity loss [39]. However, referrals to a psychologist are made through the general practitioner, sometimes requiring self-payment depending on the referral reason [9, 45]. This can act as a financial barrier for citizens with low SES, who not only have greater rehabilitation needs [7] but also more frequently experience unmet psychological needs [19–21]. Additionally, our findings reveal that fear of recurrence exacerbates anxiety and negatively impacts self-perception and outlook. Other research confirms that fear of illness hampers coping with disease or treatment consequences [28]. Our findings added that fear intensifies before routine check-ups. These check-ups occur regularly over a 5-year period post-treatment [9], heightening the long-term psychological burden and coping difficulties. This highlights the need for psychological support to cope with the psychological consequences, especially before check-ups when fear of recurrence intensifies.

## Strengths and limitations

The process of data collection and analyses are transparent by being underpinned with quotes from the participants to demonstrate the relationship between data and interpretation. A strength is that there have been two authors with different professions (occupational therapist and dental hygienist) analyzing the data, which increases credibility. Furthermore, it is a strength that the third author has contributed to the data analysis, challenging the primary authors pre-understanding. The triple-based analysis acted as a strength in enhancing in-depth understanding. Another strength is that participants were recruited from both municipal and regional settings. However, a weakness is that the study only includes participants from a municipality with specialized HNC offerings, excluding those from municipalities with limited resources and inadequate HNC interventions, which could have provided nuanced insights into rehabilitation needs. Furthermore, it constitutes a limitation that SES has not been accounted for in the data collection and data processing. This could have contributed to a deeper understanding of the rehabilitation needs, as literature on the significance of SES is incorporated in the critical interpretation.

## Clinical implications

The findings of this study add to a deeper understanding of the severe consequences and the importance of offering municipal rehabilitation to support citizens treated for HNC and thereby contribute to focus on the needs within this group of citizens and the importance of rehabilitation. Our findings underscore the importance of psychological support and interpersonal contact and access to information about others’ experiences in the rehabilitation of citizens treated for HNC. Based on previous research showing the characteristics of HNC demographics [6, 7, 18], it could be relevant to focus on citizens with low SES to prevent social inequality in health.

## Conclusion

The findings of this study contribute to an understanding of the citizen perspective on rehabilitation needs and can be used to inform and strengthen municipal rehabilitation for citizens treated for HNC. However, the findings also indicate the need for understanding the significance of health literacy, including the importance of health professionals tailoring dialog with the citizen to adapt communication and dissemination to the individual’s needs and prerequisites for handling health-related information in connection with rehabilitation. Therefore, future research should focus on the extent to which insight into the citizen’s health literacy can inform the basis for organizing municipal rehabilitation interventions, from which informational materials and guidance can be tailored to each citizen’s needs for information on managing consequences. Furthermore, the study shows the complexity of the referral process highlighting the need for further research on barriers and facilitators to referral and access to municipal rehabilitation.

## Data Availability

No datasets were generated or analysed during the current study.
